# Interaction between pristine nC_60_ and bovine serum albumin by fluorimetry: assessment of inner filter effect corrections

**DOI:** 10.3389/fbioe.2025.1518698

**Published:** 2025-02-20

**Authors:** Yu Zhang, Ye Zheng, Yuanjie Li, Shufang Liu

**Affiliations:** School of Public Health, Cheeloo College of Medicine, Shandong University, Jinan, Shandong, China

**Keywords:** fullerene, C60, inner filter effect (IFE), bovine serum albumin (BSA), mathematical correction

## Abstract

**Introduction:**

Fluorescence spectrometry is widely used to investigate nanomaterial-protein interactions, a crucial component of nanomaterial safety evaluation. However, the inner filter effect (IFE) significantly distorts fluorescence data during the analysis of fullerene (nC_60_) -protein interactions. Systematic correction methods for this system are rarely reported.

**Methods:**

In this study, bovine serum albumin (BSA) served as the protein model, four mathematical formulas (Lakowicz, Gauthier, Tucker, and Chen models) were comparatively evaluated for IFE correction in fluorescence analysis. The correction results were compared to propose an optimal correction method for the interaction between nC60 and BSA. Binding parameters were calculated from corrected data, and quenching mechanisms were analyzed using Stern-Volmer equations.

**Results:**

At room temperature with low nC_60_ concentrations (<2.0 × 10^−5^ mol/L), Chen’s model demonstrated optimal IFE correction accuracy. Corrected data indicated static quenching between nC_60_ and BSA, with a binding constant of K = 2.95 × 10^9^ L/mol and approximately two binding sites.

**Discussion:**

This study offers methodological guidance for IFE correction and accurate fluorescence analysis in the investigation of interactions between nanomaterials and biomolecules. Thus, it provides a reliable analytical method for the bio-safety assessment of nanomaterials.

## 1 Introduction

The interaction of nanomaterials with biomolecule such as protein is an important part in the safety evaluation of nanomaterials. Among the virous methods for investigating this interaction, fluorimetry is a common and widely used technique, among which the fluorescence quenching method is extensively employed in chemical and biochemical quantitative analysis due to its high sensitivity (Lakowicz, 2006). It provides information such as the quenching mechanism, quenching constant, number of binding sites, type of binding force in the interaction and distance between the fluorescence donor and acceptor. An important and unavoidable issue in fluorescence quenching analysis is the absorption of the quencher at the excitation and/or emission wavelengths of the fluorophore, which leads to a decrease in fluorescence intensity. This phenomenon is known as the inner filter effect (IFE) (Parker and Rees, 1962). The IFE can be divided into two types: the primary inner filter effect (pIFE), which arises from the absorption of the incident light, and the secondary inner filter effect (sIFE), which is caused by the absorption of the emitted fluorescence (Kubista et al., 1994). The presence of IFE often results in an inflated quenching rate, significantly impacting the determination of the quenching mechanisms between fluorophore and quencher, as well as the calculation of their binding parameters. Therefore, to accurately evaluate the biosafety of nanomaterials, it is crucial to distinguish between the IFE and the actual quenching effect and to make appropriate adjustments to avoid substantial errors in the calculation of binding parameters.

To address this challenge, numerous researchers have developed various approaches to rectify the IFE(Ceresa et al., 2021; Kimball et al., 2020; Kumar Panigrahi and Kumar Mishra, 2019; Wang et al., 2017). Both physical and chemical methods for IEF correction (Kubista et al., 1994; Tobias et al., 2007) encounter significant challenges, such as complexity and high costs, which limit their widespread adoption. In contrast, mathematical correction methods are convenient, rapid, and cost-effective, making them widely employed in the correction of fluorescence across a range of substances (Holland et al., 1977; Puchalski et al., 1991). In reports on the interaction between proteins and small molecules or other exogenous substances, many have directly ignored the influence of IFE (Banerjee and Das, 2013; Guan et al., 2018; Yan et al., 2013; Zhang et al., 2023), while most of the remaining studies have chosen the simplest equation designed by Lakowicz for IFE correction (Rahman et al., 2021; Tang et al., 2012). However, the effectiveness of different mathematical formulas varies in correcting inner filter effects, The applicability of different calculation formulas varies, and each formula has specific prerequisites for application. Relying on a single equation for correction may lead to discrepancies in the results.

Fullerenes, as an important carbon nanomaterial, have been extensively studied for their biosafety, including their interactions with proteins. Previous studies have utilized a diverse array of methods to explore the interactions between fullerenes and various types of proteins (Liu et al., 2021; Cai and Chen, 2019; Gaponenko et al., 2020; Wan et al., 2020). When analyzing these interactions through fluorescence quenching techniques (Bai et al., 2021; Gupta et al., 2011; Wu et al., 2023), the IFE is nearly inevitable, which is likely to lead to inaccuracies in the calculation of the quenching data. However, the application of fluorescence inner filter corrections for fullerene and protein has rarely been reported.

Based on the above considerations, in this study, nC_60_ was selected as the representative of nanomaterials, and BSA was served as the protein model. The interaction between BSA and nC_60_ was investigated by spectroscopic methods, with the fluorescence inner filter effect fully considered. To simplify the IFE correction process, four common mathematical methods were used for IFE correction, so as to prefer one applicable to the BSA-nC_60_ system. This work may propose reference for future research on the IFE correction of interactions between nanomaterials including carbon nanomaterials and proteins by fluorescence method and thus offer a reliable tool for the biosafety evaluation of nanomaterials.

## 2 Materials and methods

### 2.1 Materials

C_60_ (99.9%, powder) was purchased from Henan Fullerene Nano New Materials Technology Co. Ltd. (China). BSA (free fatty acid fraction V) and phosphate buffer saline (PBS) premixed powder were purchased from Solarbio (United States). Toluene (analytically pure) was purchased from Tianjin Ding Sheng Xin Chemical Co. Ltd. (China). All the dispersions and solutions were prepared with ultra-pure water. All experiments were performed at room temperature.

### 2.2 Preparation of nC_60_ aqueous dispersion

0.05 g of C_60_ powder was dissolved in approximately 5 mL of toluene and then 300 mL ultra-pure water was added to the purple solution. The mixture was subjected to ultrasonic treatment continuously for 24 h, resulting in a brown-yellow nC_60_ aqueous dispersion. Then removed toluene layer and placed it in a fume hood as long as the residual toluene was fully evaporated. The solution was preserved at room temperature, away from light. The concentration of nC_60_ was determined as 2.45 × 10^−5^ mol/L, according to the method described (Deguchi et al., 2001).

Imaging of nC_60_ was performed by HT-7700 transmission electron microscope (TEM) (Hitachi, Japan) and JEM-1011 TEM (JEOL, Japan).

### 2.3 UV-Vis spectroscopy measurements

The absorption spectra of nC_60_ dispersion and BSA-nC_60_ systems were measured by U2900 UV-Visible spectrophotometer (Hitachi, Japan) with a 10 mm pat length cuvette.

### 2.4 Fluorescence spectroscopy studies

The Fluorescence measurements were performed on F4700 fluorescence spectrophotometer (Hitachi, Japan) with a 10 mm path length quartz cuvette. The excitation wavelength ( λex ) of the protein and the mixture solution was set at 280 nm, and the emission spectra of BSA (1.0 × 10^−5^ mol/L) were recorded in the absence or presence of nC_60_ with different concentrations in the wavelength range of 300∼550 nm. All measurements were repeated in triplicate.

Fluorescence lifetime was measured using FLS-920 steady-state/time-resolved fluorescence spectrometers (Edinburgh, England). The samples were excited at 280 nm using nanosecond diode excitation source.

### 2.5 Mathematical correction of fluorescence IFE

For the obtained BSA-nC_60_ mixed system, the following four common mathematical models were chosen to correct the fluorescence data:

$$F_{corr}=F_{obs}\times 10^{\left(A_{ex}+A_{em}\right)/2}$$ (1)

Equation 1 represents the Lakowicz’s model (Lakowicz, 2006). $F_{corr}$ is the corrected fluorescence intensity using the IFE equation, $F_{obs}$ denotes the observed fluorescence intensity, and $A_{ex}$ and $A_{em}$ are the absorbance of BSA-nC_60_ mixed system at the excitation and emission wavelengths, respectively.

$$\frac{F_{corr}}{F_{obs}}=\frac{2.3{03A}_{ex}\left(y-x\right)}{\left(10^{-xA_{ex}}-10^{-yA_{ex}}\right)}\times \frac{2.3{03A}_{em}\left(v-u\right)}{\left(10^{-uA_{em}}-10^{-vA_{em}}\right)}$$ (2)

**FIGURE 1:**
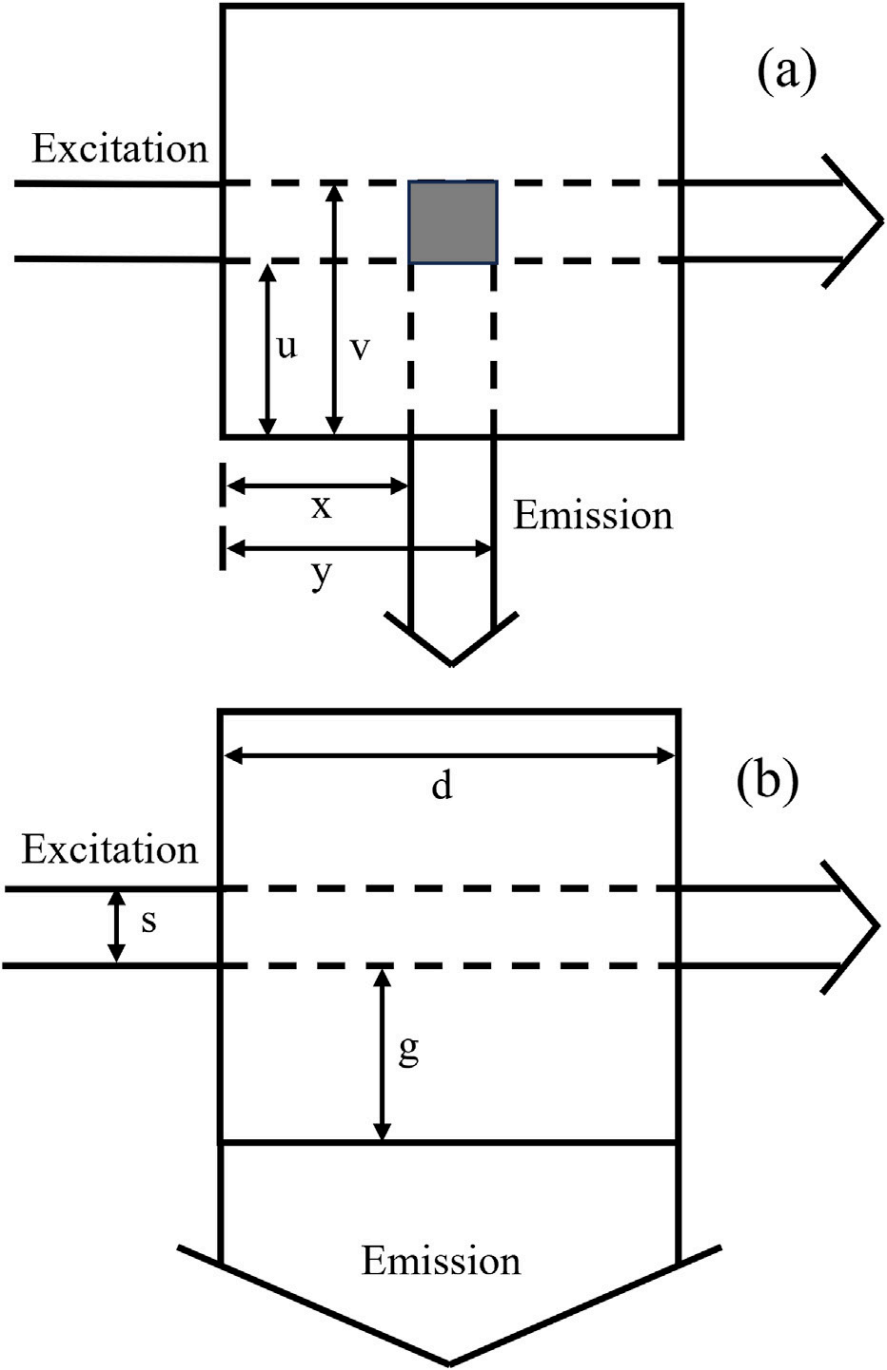
Cell geometry parameters from the top view: **(A)** Tucker’s model. parameters (x, y) and (u, v) are determined by masking apertures in emission and excitation beam, respectively. **(B)** Gauthier’s model. s is the excitation beam width, g is the distance between the beam and the cuvette, d is the width of the cuvette.

Equation 2 represents the Tucker’s model (Tucker et al., 1992). The parameters *x*, *y*, *u*, and *v* are distances specific to cell geometry depicted in Figure 1A. In this experiment, x = 0.1, y = 0.8, u = 0.1, and v = 0.8. All geometrical parameters are in centimeters.

$$\frac{F_{corr}}{F_{obs}}=\frac{2.3dA_{ex}}{1-10^{-dA_{ex}}}\times 10^{g\cdot Aem}\times \frac{2.3sA_{em}}{1-10^{-sA_{em}}}$$ (3)

Equation 3 represents the Gauthier’s model (Gauthier et al., 1986). d is the pathlength of the quartz cuvette (generally 1 cm), and s and g are cell geometry parameters related to the exciting and emitting beams, illustrated in Figure 1B. In this experiment, d = 1, s = 0.7, g = 0.1.

$$F_{corr1}=\frac{\left(1+10^{-A_{2}/n}\right)\left(1-10^{-A_{1}/n}\right)\left[1-10^{-\left(A_{1}+A_{2}\right)}\right]}{2\left(1-10^{-A_{1}}\right)\left[1-10^{-\left(A_{1}+A_{2}\right)/n}\right]}\times F_{obs1}$$ (4)

$$F_{corr2}=10^{A_{\left(\lambda em\right)}/2}\times F_{obs2}$$ (5)

**FIGURE 2:**
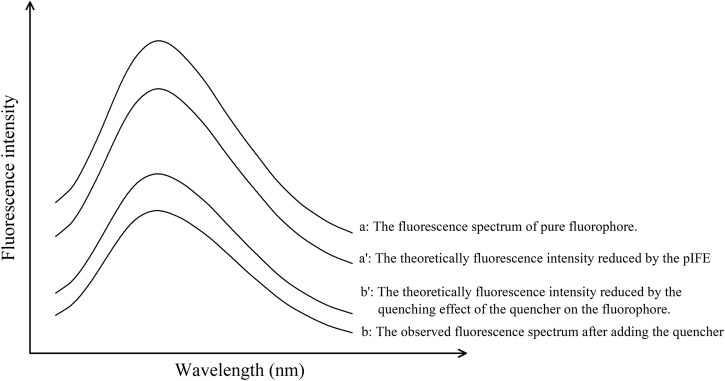
Theoretical fluorescence quenching spectra in an ideal state according to Chen’s model. This method is universally applicable to all fluorescent substances, hence the x-axis is labeled as “Wavelength (nm)” without specific values, ensuring broad applicability and generalization across various fluorescent materials.

Equations 4, 5 represent the Chen’s model (Chen et al., 2015), Figure 2 illustrated the theoretical concept diagram of Chen’s model, since this method is universally applicable to all fluorescent substances, the x-axis is labeled as ‘Wavelength (nm)’ without any specific values. Referring to Figure 2 and Equations above, $F_{obs1}$ is the fluorescence intensity of the pure fluorophore from curve a, $F_{corr1}$ is the corrected result of pIFE from curve a’, $A_{1}$ and $A_{2}$ are the absorption data of fluorophore and quencher at excitation wavelengths, respectively. n is the theoretical layer number (usually equals to 100 or 1,000, in this experiment, n = 1,000), $F_{obs2}$ is the fluorescence intensity with quencher from curve b, $F_{corr2}$ is the corrected result of sIFE from curve b’, $A_{\left(\lambda em\right)}$ is the absorption data of quencher at emission wavelength.

## 3 Results and discussion

### 3.1 Determination of the IFE in BSA-nC_60_ fluorescence quenching system

**FIGURE 3:**
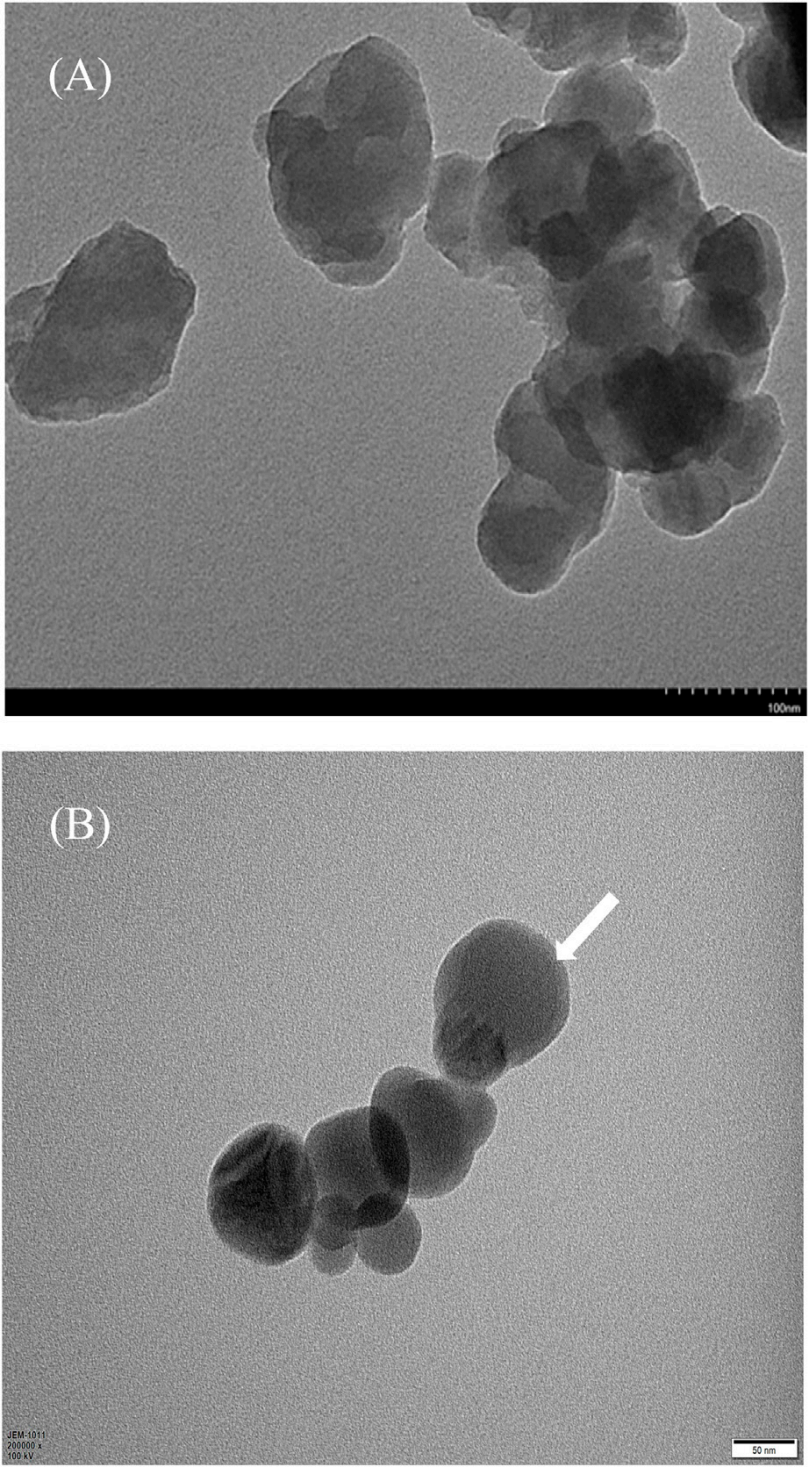
**(A)** TEM image of nC_60_ nanoparticles. **(B)** TEM image of nC_60_ combined with BSA. (write arrow indicate the adsorption of BSA on the surface of nC_60_).

Transmission Electron Microscopy (TEM) provides an intuitive means to observe the shape, size, and distribution of nanoparticles. Figure 3A showed the TEM image of nC_60_ aqueous dispersion after drying, as observed, nC_60_ nanoparticles exhibited irregular circular shapes and aggregated together, with a particle size ranging approximately between about 50 and 100 nm. And Figure 3B showed that, after adding BSA, the surface of nC60 was covered with a slightly transparent protein corona, and the overall morphology became more regular. This is consistent with the reported adsorption of proteins onto C60 nanoparticles in the previous studies (Deguchi et al., 2007; Wu et al., 2011; Vance et al., 2016).

**FIGURE 4:**
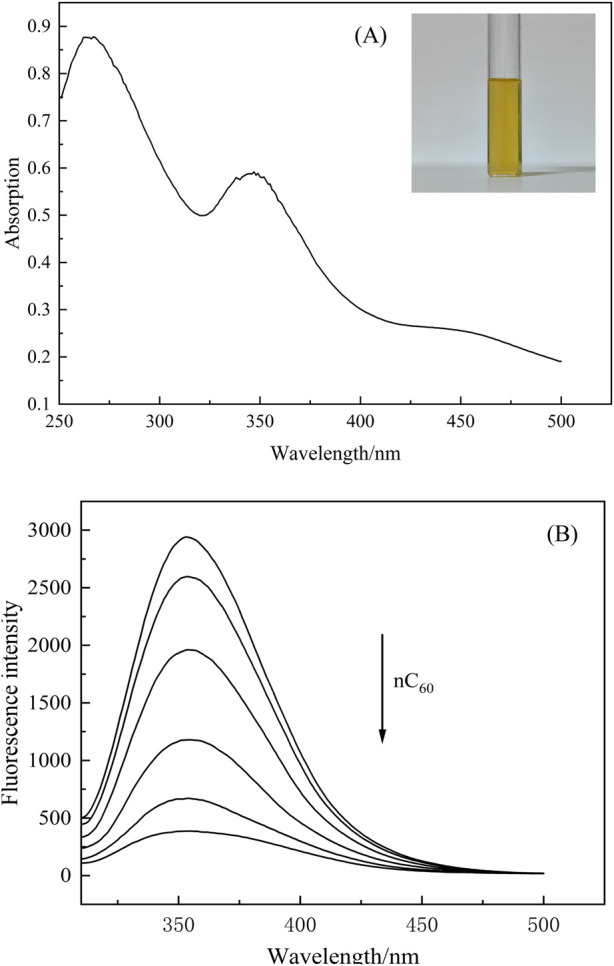
**(A)** Absorption spectrum of nC_60_ (the concentration of nC_60_ is 2.45 × 10^−5^ mol/L); **(B)** Fluorescence spectra of BSA (1.0 × 10^−5^ mol/L) with nC_60_. (concentrations of nC_60_ from up to down were 0.00, 1.96, 4.90, 9.81, 15.4 and 19.6 × 10^−6^ mol/L; pH = 7.4; $\lambda_{ex}$ = 280 nm).

Previous studies have already found that nC_60_ has a quenching effect on proteins (Li et al., 2013; Noskov et al., 2021), but the influence of IFE has rarely been considered. In order to confirm there is an inner filter effect in BSA-nC_60_ fluorescence system, the absorption spectra of nC_60_ aqueous dispersion were measured in the range of 250–550 nm which was shown in Figure 4A. Figure 4A revealed that nC_60_ aqueous dispersion had two notable absorption peaks at 270 nm and 354 nm, the latter being characteristic of C_60_ nanoparticles. The fluorescence spectra of the BSA-nC_60_ solution are shown in Figure 4. Combining Figure 4A, B, it can be observed that the absorption of nC_60_ overlaps with the excitation (280 nm) and emission (350 nm) wavelengths in the fluorescence spectra of the subsequent solution. This overlap suggested that the mixed solution of nC_60_ and BSA could had experienced some degree of interference from the fluorescence IFE during measurements.

### 3.2 Selection of the correction methods for the IFE in nC_60_-BSA system

IEF is hard to avoid in right-angle fluorometry. In order to ensure that the quenching effect of nC_60_ on BSA is not solely caused by IFE, it is necessary to correct it. Researchers have proposed various mathematical models for different situations (Lakowicz, 2006; Tucker et al., 1992), and the effectiveness of the correction varies accordingly. Therefore, selecting an appropriate mathematical formula is crucial. For the BSA-nC_60_ system, the four aforementioned mathematical models were applied for calibration. All the experimental dates required for calibration are listed in Table 1, and the calibration results are shown in Table 2. Then the fluorescence quenching dates were analyzed by the Stern-Volmer equation (Zhang et al., 2023).

$$F_{0}/F=k_{q}\tau_{0}\left[Q\right]+1=K_{SV}\left[Q\right]+1$$ (6)

**TABLE 1:** Fluorescence emission intensity and absorbance data for BSA and various concentrations of nC_60_.

C_nC_60__ (mg/mL)	*Fobs*	*Aex* (280 nm)	*Aem* (350 nm)	*A1*	*A2*	*A(λem)*
0.00 × 10^−6^	2,938.5	0.404	0.009	0.404		
1.96 × 10^−6^	2,595	0.469	0.06		0.064	0.051
4.90 × 10^−6^	1963	0.565	0.136		0.160	0.127
9.81 × 10^−6^	1,177.7	0.725	0.263		0.320	0.253
1.54 × 10^−5^	667.7	0.885	0.390		0.481	0.381
1.96 × 10^−5^	385.8	1.046	0.517		0.642	0.508

**TABLE 2:** Corrected Fluorescence emission intensity for BSA and various concentrations of nC_60_.

*C*_*nC60*_ * (mg/mL)*	*F*_*corr*_ *by Tucker*	*F*_*corr*_ *by Gauthier*	*F*_*corr*_ *by Lakowicz*	*F*_*corr1*_ *by Chen*	*F*_*corr2*_ *by Chen*
0.00 × 10^−6^	4,575.23	4,549.01	4,730.81		
1.96 × 10^−6^	4,623.23	4,501.95	4,768.78	2,762.73	2,751.23
4.90 × 10^−6^	4,197.48	4,022.95	4,399.27	2,526.69	2,271.93
9.81 × 10^−6^	3,382.53	3,150.95	3,674.05	2,195.42	1,577.56
1.54 × 10^−5^	2,547.29	2,301.22	2,899.62	1926.62	1,035.16
1.96 × 10^−5^	1933.94	1,691.10	2,332.23	1706.59	692.25

In Equation 6, F_0_ and F represent the fluorescence intensity of BSA in the absence and presence of different concentrations of nC_60_ respectively. τ_0_ is the average fluorescence lifetime of biomacromolecules without quencher. k_q_ is the dynamic quenching rate constant (L mol^−1^ s^−1^) and K_SV_ is the dynamic quenching constant (L/mol). [Q] refers to the concentration of the quencher.

**FIGURE 5:**
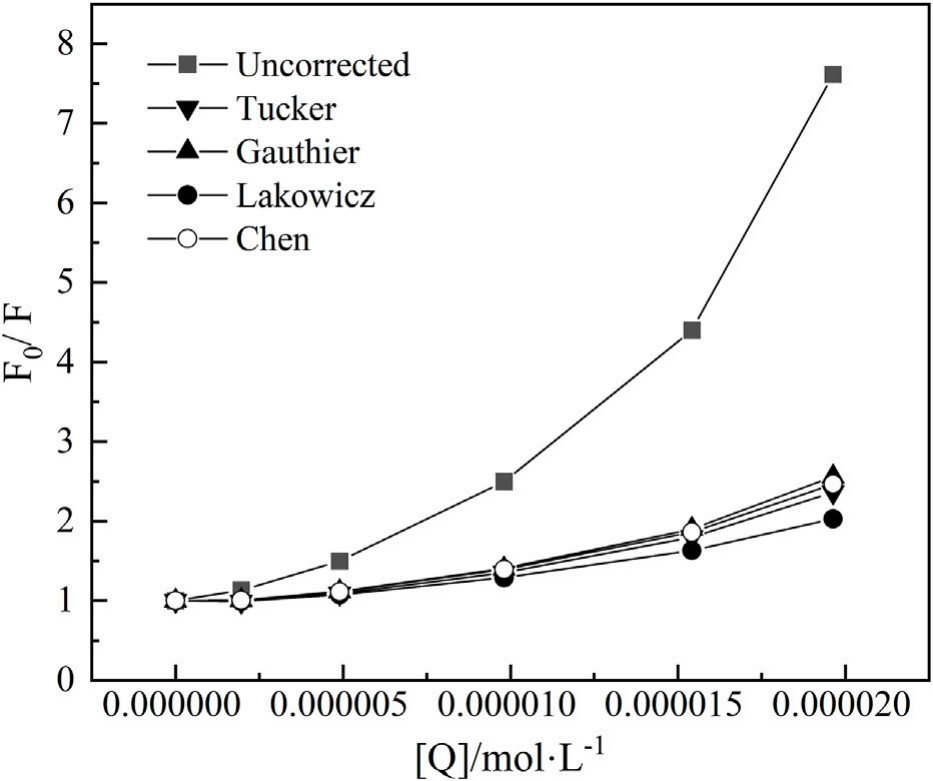
The plots for the fluorescence quenching of BSA by nC_60_ at $\lambda_{ex}$ = 280 nm. ([Q] is the concentration of nC_60_).

Figure 5 displayed the fluorescence quenching plots of BSA by nC_60_. It showed that the quenching curve obtained without correcting the IFE was not linear but curved to the Y-axis as the quencher concentration increased. After correction, the degree of quenching significantly decreased, about 67.5% lower than before, indicating that all four correction methods can effectively reduce the influence of the IFE. So further analysis is required to assess which model was more suitable in this study. The conditions for using each model are described in detail as follows.

Firstly, the Lakowicz model is the simplest method and is therefore widely used. However, this is an approximate method, as its theoretical basis assumes that the emission radiation is observed at the center of a 1 cm path length cuvette, and it may not be true since the geometry of the sample compartment is different. An additional drawback is that this model is valid only up to A = 0.7, according to Panigrahi and Mishra (2019). For larger values of A, this model overestimates the loss of observed fluorescence due to IFE, resulting in an upward curvature of the corrected fluorescence. However, the absorbance of this experimental system is relatively high (up to 1.045), so applying the Lakowicz model may lead to over-correction.

Tucker’s model and Gauthier’s model are both based on different sample geometries (dependent on the fluorescence light collection position) and the absorption characteristics of the solution in a vertical monochromator slit system for correction. Yet the Hitachi fluorescence spectrophotometer used in this experiment employs a relatively uncommon horizontal monochromator slit system. In Gauthier’s model, the excitation beam with thickness s = 0.10 cm, and the distance between the beam to the cuvette g = 0.40 cm (Gauthier et al., 1986). However, in this experimental apparatus, the actual measured value of s = 0.7 cm, and g = 0.1 cm the different measurement of geometric factors may impact the final correction.

More importantly, in the calibration of pIFE, the three models mentioned above are predicated on the assumption that the emitted fluorescence is proportional to the entire system’s absorption of the excitation light. However, in a mixed system where a quencher is present, only the protein emits fluorescence, while both nC_60_ and the protein absorb light. This results in a decrease in the effective absorbance of the protein, potentially leading to over-correction in the calculations of these models.

To address this limitation, Chen proposed a novel theoretical model based on an in-depth study of the physical mechanisms of IFE, which does not depend on geometric parameters. Theoretically, the observed fluorescence intensity is proportional to the effective absorption of excitation light (EAEF) by the fluorophore, which can be calculated using the Beer-Lambert law. In mixed solutions, the competition for absorption from quenching agents can modify the EAEF compared to pure solutions, complicating direct calculations.

Chen’s approach employs a layered method that divides the solution in the sample cell into n equal thin layers, calculating the absorbance of each layer step by step. This layered approach effectively addresses the interactions among components in mixed solutions, allowing for a more accurate calculation of transmitted light intensity. The number of layers in this method influences the calculation’s accuracy; specifically, when/n < 0.01, the error in Chen’s model is reduced to less than 1.2% (Chen et al., 2015).

In Figure 5, the first corrected dots of the Lakowicz’s and Tucker’s model showed F_0_/F < 1. This indicated that, after correction, the fluorescence intensity of BSA with the addition of nC_60_ exceeded that of pure BSA. It suggested that both the Lakowicz and Tucker models over-corrected the fluorescence intensity of the BSA-nC_60_ mixed system. In contrast, the Gauthier’s and Chen’s models were more suitable for this system. Considering that Chen’s model does not involve the effects of geometric factors and accurately accounts for the effective absorbance of excitation light, Chen’s model was ultimately selected for further study. Nevertheless, the appropriateness of this model needs to be further confirmed based on the calibration results to ensure its suitability compared to other potential models.

### 3.3 Comparison of fluorescence analysis results before and after IFE correction

The mechanism of fluorescence quenching is categorized into static quenching, dynamic quenching, and combined dynamic and static quenching (Noskov et al., 2021), which requires the selection of appropriate formulas for different quenching mechanisms when calculating the interactions of small molecules and proteins with binding sites and binding constants. The Stern-Volmer plot of dynamic quenching alone or static quenching with a binding site of 1 should be a straight line with an intercept close to 1, whereas the quenching curve curves toward the Y-axis in the case of combined dynamic-static interaction or static quenching with multiple binding sites.

Considering that the corrected quenching curves in Figure 5 still curved to the longitudinal axis, it can be preliminarily concluded that the mechanism for the quenching of BSA by nC_60_ was likely to be multisite static quenching or dynamic and static combined quenching. Firstly, assumed that the quenching mechanism involved static quenching at multiple binding sites, which can be represented by Equation 7 (Song et al., 2011):

$$F_{0}/F=K{\left[Q\right]}^{n}+1$$ (7)

where K is the formation constant of the fluorophore-quenching agent complex (L/mol), *n* is the number of binding sites. [Q] is the concentration of nC_60_. The binding parameters obtained from Equation 6 were presented in Table 3.

**TABLE 3:** Parameters of the static quenching of multiple binding sites.

*Correction method*	*n*	*K (L/mol)*	*R* ^*2*^
Uncorrected	2.30	4.41 × 10^11^	0.992
Tucker’s modle	2.04	5.58 × 10^9^	0.998
Gauthier’s model	2.03	5.40 × 10^9^	0.998
Lakowicz’s model	1.90	9.13 × 10^8^	0.999
Chen’s model	1.98	2.95 × 10^9^	0.998

Secondly, assuming that the fluorescence quenching of BSA by nC_60_ was dynamic and static combined quenching. The Equation for dynamic and static quenching is depicted in Equation 8 (Liu et al., 2021), and the binding parameters obtained using Equation 8 were shown in Table 4.

$$F_{0}/F=K_{SV}{\left[Q]+K[Q\right]}^{n}+K_{SV}K{\left[Q\right]}^{n+1}+1$$ (8)

**TABLE 4:** Parameters of the dynamic and static combined quenching.

*Correction method*	*K*_*SV*_ *(L/mol)*	*K (L/mol)*	*n*	*R* ^*2*^
Uncorrected	102423.77	2.01 × 10^14^	3	0.999
Tucker’s modle	5,612.14	1.01 × 10^10^	2.11	0.998
Gauthier’s model	14476.71	3.92 × 10^10^	2.25	0.998
Lakowicz’s model	−651.27	8.69 × 10^8^	1.89	0.988
Chen’s model	12344.48	1.32 × 10^10^	2.15	0.998

In Equation 8, $K_{SV}$ is Stern-Volmer quenching constant. K is the formation constant of the fluorophore-quenching agent complex (L/mol). *n* is the number of binding sites.

According to Table 4, if the quenching was a combination of dynamic and static quenching, in the correction results of Lakowicz’s model, the fitted $K_{SV}$ was negative, while the dynamic quenching rate constant should be positive, which further demonstrates that Lakowicz’s model was not applicable to this system.

Comparing Tables 3, 4, it can be seen that regardless of whether the quenching mechanism was static quenching at multiple sites or a combination of dynamic and static quenching, the calculated results of Gauthier and Chen’s models were close to each other, and the number of binding sites were around 2, suggesting that there may be two potential binding sites for nC_60_ with BSA. However, even under the assumption of a dynamic combined static quenching mechanism, the value of k_q_, calculated using Equation 6 based on τ_0_ (generally 10^−8^ s) and the K_SV_ data presented in Table 4, yielded a value of 1.23 × 10^12^ L mol^−1^ s^−1^, which significantly exceeds the maximum scatter collision quenching constant of various quenchers (2 × 10^10^ L mol^−1^ s^−1^). This result suggested that the quenching mechanism is predominantly static in nature.

Merely comparing the results from the four correction formulas in Tables 3, 4 does not allow us to determine which assumption is more reasonable. To obtain precise binding information, it is essential to ascertain the proportion of static quenching involved.

### 3.4 Fluorescence lifetime of BSA in the absence and presence of nC_60_

Time-resolved fluorescence technology enables the determination of the fluorescence lifetime of biological macromolecules. To further confirm the quenching mechanism of BSA-nC_60_ system, the fluorescence lifetimes of BSA were measured before and after the addition of nC_60_. Theoretically, dynamic quenching typically increases the quenching effect with increasing concentration, leading to a significant shortening of the fluorescence lifetime. In contrast, static quenching tends to have minimal changes in fluorescence lifetime (Mohanty and Subuddhi, 2022).

**FIGURE 6:**
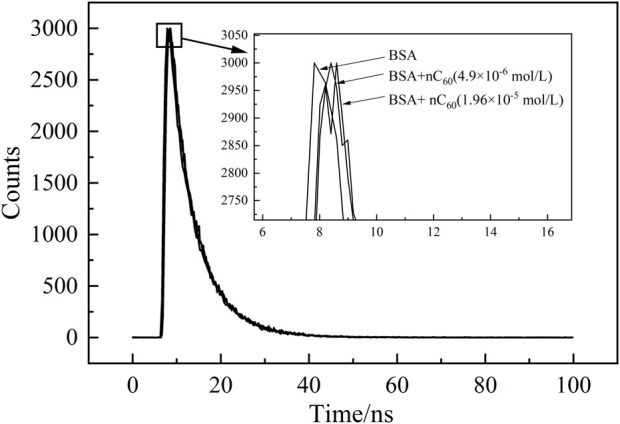
Time-resolved fluorescence spectra of BSA (1.0 × 10^−5^ mol/L) in the absence and presence of nC60.

The time-resolved fluorescence spectra of the system were shown in Figure 6, where the vertical axis represents the number of detected photons and the horizontal axis represents time. By fitting the fluorescence decay curve obtained from the time-resolved spectrum, the fluorescence lifetime (τ) of BSA was measured to be 6.13 nanoseconds in the absence of nC_60_. Following the addition of varying concentrations of nC_60_, the fluorescence lifetimes were observed to shift to 6.06 nanoseconds and 6.14 nanoseconds, respectively. The results indicated that although there were variations in these τ values, these minor differences can be attributed to photon scattering, as the fluorescence lifetime changes are minimal and do not deviate significantly from single-exponential decay. In other words, the quenching of BSA by nC60 did not significantly alter the fluorescence lifetime of BSA, further supporting that the quenching mechanism of BSA-nC_60_ is primarily static quenching. Therefore, it is more reasonable to use the data from Table 3 for subsequent data analysis.

Given that the Chen’s model is immune to geometric factors and absorbance, and considers the effective absorbance of both the protein and the quencher, it was ultimately selected for the binding analysis. This model is suitable for interpreting the fluorescence data of BSA in the presence of low concentrations of nC_60_. The corrected fitted curve demonstrated a significant decrease in the upward trend when compared to the uncorrected data, which indicates a substantial reduction of the fluorescence inner filter effect. Through the integration of fluorescence lifetime measurements, it was established that the BSA-nC_60_ system undergoes static quenching, characterized by a binding constant of 2.95 × 10^9^ L/mol and approximately two binding sites. This methodology provides a more precise evaluation of the influence of nC60 on the fluorescence attributes of BSA, allowing researchers to gain deeper insights into the underlying molecular mechanisms. While this method has demonstrated its effectiveness in BSA and nC60 system, it is essential to acknowledge that its applicability to other materials and proteins remains unverified. To fully establish the general utility of this approach, future research should focus on expanding its application to a broader range of systems, including various proteins and nanomaterials. Moreover, due to the unique physicochemical properties of nanomaterials, the model needs further adjustments to achieve higher accuracy. The outcomes of this study will not only offer comprehensive insights into the interaction between nanomaterials and biomolecules but also provide foundational support for future investigations into nanomaterials biosafety.

## 4 Conclusion

The fluorescence quenching effect between bovine serum albumin (BSA) and nC_60_ is significantly influenced by inner filter effects (IFE), therefore, it is imperative to select an appropriate correction model specifically tailored for the nC_60_-BSA system in order to prevent misinterpretation of analytical results. In this study, we thoroughly examined four common IFE correction models and identified the most suitable one (Chen’s model) for systems involving high absorbance nanomaterials and protein quenching. Following the application of these corrections, we investigated the interaction mechanism between BSA and nC_60_ and further obtained the binding parameters and the number of binding sites. This research established a theoretical foundation for the accurate calculation of quenching information and binding data in analogous quenching systems, thereby providing a methodological reference for the study of interactions between nanomaterials and other exogenous substances or proteins. However, given the unique physical and chemical properties of nanomaterials, which are capable of both absorbing and significantly scattering light, future studies should integrate scattering effects into the IFE correction to enhance its applicability to carbon nanomaterials.

## Data Availability

The original contributions presented in the study are included in the article/supplementary material, further inquiries can be directed to the corresponding author.
